# Inventory Control Mechanism of the Pharmacy Store of a Recently Established National Institute in Eastern India: A Cross-Sectional, Investigative Analysis

**DOI:** 10.7759/cureus.49632

**Published:** 2023-11-29

**Authors:** Rishabh Jaju, Saurabh Varshney, Pratima Gupta, Pooja Bihani, Habib Md R Karim

**Affiliations:** 1 Anaesthesiology and Critical Care, AIl India Institute of Medical Sciences, Deoghar, Deoghar, IND; 2 Otorhinolaryngology, All India Institute of Medical Sciences, Deoghar, Deoghar, IND; 3 Microbiology, All India Institute of Medical Sciences, Deoghar, Deoghar, IND; 4 Anaesthesiology, Dr. Sampurnanand Medical College, Jodhpur, IND; 5 Anesthesiology and Critical Care, All India Institute of Medical Sciences, Deoghar, Deoghar, IND

**Keywords:** abc-ved matrix, ved analysis, abc analysis, inventory management, pharmacy

## Abstract

Background

New establishments frequently face challenges. Pharmacy is integral to healthcare delivery institutes, and inventory management is crucial. The present study investigated the problems faced by the pharmacy of a newly established health institute and proposed relevant solutions to identify drugs needing stringent management control.

Methods

Responses were collected from seven pharmacists for questionnaires focusing on pharmacy problems and possible solutions. Always better control (ABC), vital essential desirable (VED), and ABC-VED matrix analysis were done on the drugs dispensed during the financial 2022-23.

Results

The predominant challenges identified were stockouts and shortages, expiry of medications, and supplier-related issues. The causes were mainly related to rural location, communication hurdles, and vendor management. Proposed solutions were integrating patients' electronic health records and bar code technology with the pharmacy's inventory management system, conducting pharmacy knowledge and skills upgradation sessions every six months, adopting ABC and VED analysis, and first-in first-out (FIFO), just-in-time (JIT). Total annual drug expenditure (ADE) for the drugs dispensed was 1,18,81,520 Indian Rupees. ABC analysis revealed 109 (22.8%), 115 (24.06%), and 254 (53.14%) of medicines as A, B, and C categories, respectively, accounting for 69.98, 20.00, and 10.07% of ADE. On ABC-VED matrix analysis, 125 (26.15%), 267 (55.86%), and 86 (17.99%) of drugs were found to be category I, II, and III items, respectively, accounting for 71.52, 23.84, and 4.64% of ADE.

Conclusion

Our study identified different pharmacy problems for a developing institute and suggested relevant measures. Categorization of drugs based on ABC and VED analysis will help to strengthen inventory control.

## Introduction

It is well known that about one-third of the annual hospital budget is spent buying materials and supplies, including medicines [1,2]. A pharmacy is one of a hospital's most extensively used therapeutic facilities, regularly consuming significant money on purchases. Pharmacies face numerous challenges regarding inventory handling, manual inventory management, inaccurate demand forecasting, lack of real-time visibility, and expiry management [3]. Inventory management can guide us in better planning and managing the supply chain of the items that require greater attention effectively [2].

For material management, always better control (ABC) and vital essential desirable (VED) analytical methods are commonly used [4]. ABC analysis follows Pareto's principle of "separating the vital few from the trivial many" based on the capital investment of the item. Drugs are prioritized according to their clinical and economic impact based on their cost and volume. VED analysis is based on critical values and the deficiency cost of the items. Combining ABC and VED analysis (ABC-VED matrix) has proved advantageous to gain significant control over the material supplies [5].

Our institute recently started delivering outpatient department (OPD) services in August 2021 and inpatient department (IPD) and operation theatre (OT) services from July 2022. Therefore, the patient load served is minimal compared to other full-fledged institutes in our country. Nevertheless, it is a tertiary care hospital located in a rural area with no tertiary care hospitals within a radius of 100 km. Most patients served by our institute belong to lower socio-economic groups and depend significantly on the subsidized and free healthcare services provided by the public sector set-ups. Rational drug use and improved drug management can aid in serving more patients efficiently by providing organized clinical and administrative services and improving patient satisfaction.

The present study aimed to assess the problems faced by pharmacists in inventory management. As a part of this project, we also conducted the first-ever ABC and VED analysis of the drugs dispensed by the pharmacy store of our institute during the year 2022-23.

## Materials and methods

With approvals from the Institute Ethical Committee (IEC), this cross-sectional, exploratory study was designed to investigate the problems faced by the pharmacy of our institute. The study involves a cross-sectional questionnaire-based assessment conducted during the third and fourth week of October 2023. Further, inventory drug orders, costs, and the nature of the drug procured and dispensed from April 2022 to March 2023 were also analyzed during the first two weeks of October 2023.

The primary investigator talked with a pharmacist with work experience in an established and newly established institute. Based on the interview, he prepared a questionnaire for inventory management-related problems (stocking, ordering, supply chain, disposal, technology, etc.) [6] and suggested possible solutions. It was followed by an internal review of the questions by all the investigators and external validation by three experienced persons in relevance, clarity, simplicity, and ambiguity; the content validity index was calculated to be one. The final questionnaire had ten semi-closed-ended questions (Appendix 1) printed and distributed to the study participants.

We approached the pharmacists employed in our institute to participate and provide the responses. The responses from these participants were collected anonymously. We included the inventory management data for drugs, but the data for medical equipment and consumables were not included in the study. Only data available from the pharmacy computer in the electronic form for inventory management were considered.

The questionnaire responses are presented in absolute number and percentage scales. To perform the ABC, VED, and ABC-VED matrix analysis, each drug formulary's annual consumption and expenditure were collected, transcribed in an MS Excel spreadsheet, and analyzed.

For ABC analysis, annual usage for each drug (consumption x cost) was calculated. Individual drugs were arranged in descending order based on their annual expenditure, and the cumulative cost of all drugs was calculated. The cumulative percentage of the number of items and the cumulative percentage of expenditure were then computed. This list was then subdivided into three categories based on the cumulative cost percentage: A (70%), B (20%), and C (10%), respectively.

For VED analysis, drugs were categorized based on their importance in clinical settings, considering the standard definition of vital, essential, and desirable drugs.

For ABC-VED matrix analysis, drugs were divided into three categories (I, II, and III) by cross-tabulating the ABC and VED analysis. All vital and expensive drugs constituted category I belong to AV, AE, AD, BV, and CV subcategories. The BE, CE, and BD subcategories constituted category II, and the remaining cheaper and desirable drugs in the CD subcategory constituted category III [7].

## Results

When conducting the survey, seven pharmacists worked in our institute, and all pharmacists responded to the questionnaire; the responses are presented in Table 1. Predominant issues were overstocking, stockouts and shortages, expiry of medications, and supplier-related; most participants agreed to the existence of multiple issues. All respondents believed there were communication hurdles, while 4 (57.1%) thought the challenges were due to cost matters. The delays leading to drug shortages were predominately related to the rural location of the institute and vendor management as per the school of thought of 6 (85.7%) and 4 (57.1%) pharmacists, respectively.

**Table 1:** Questions and responses by the participants expressed in absolute number and percentage scale. ABC: always better control, VED: vital essential desirable

Questions	Options	Responses
What challenges or issues are most frequently encountered in our hospital's pharmacy inventory management?	Overstocking	3 (42.8 %)
	Stockouts and shortages	3 (42.8 %)
	Expiry of medications	4 (57.1 %)
	Supplier-related	3 (42.8 %)
What is the leading cause of pharmacy-related issues in our hospital?	Communication breakdowns	7 (100 %)
	Costing related	4 (57.1 %)
	Technology related	1 (14.2 %)
Do you need help with stockouts/shortages in our pharmacy?	Regularly	2 (28.5 %)
	Often	3 (42.8 %)
	Occasionally	2 (28.5 %)
What do you think is the main reason for these delays?	Rural location	6 (85.7 %)
	Vendor related	4 (57.1 %)
	Product quality	1 (14.2 %)
Which inventory control method is effective in pharmacy inventory management?	ABC analysis	3 (42.8 %)
	VED analysis	2 (28.5 %)
	First-in first-out	5 (71.4%)
	Just-in-time	3 (42.8%)
How relevant is integrating bar code technology with a pharmacy's inventory management system?	Highly relevant	3 (42.8 %)
	Quite relevant	1 (14.2 %)
	Somewhat relevant	3 (42.8 %)
How relevant is integrating Patients' electronic health records with the pharmacy's inventory management system?	Highly relevant	4 (57.1 %)
	Quite relevant	3 (42.8 %)
How often should we conduct pharmacy knowledge and skills upgrade sessions for the pharmacy staff?	Every three months	1 (14.2 %)
	Every six months	6 (85.7 %)
What are the necessary training skills that we should focus on?	Inventory management techniques	5 (71.4 %)
	Documentation	3 (42.8 %)
	Software training	6 (85.7 %)
	Communication skills	4 (57.1 %)
How can doctors help improve the pharmacy management of a hospital?	Writing accurate and legible prescriptions	3 (42.8 %)
	Suggest alternative drugs during non-availability	6 (85.7 %)
	Timely and effective communication	3 (42.8 %)
	Integration of patients’ electronic health records	4 (57.1 %)

First-in first-out (FIFO); ABC analysis and just-in-time (JIT) were effective pharmacy inventory control methods as per the opinions of 5 (71.4%), 3 (42.8%), and 3 (42.8%) pharmacists, respectively. Integration of patients’ electronic health records and bar code technology with the pharmacy's inventory management system was highly relevant as per the opinions of 4 (57.1%) and 3 (42.8%) pharmacists, respectively.

According to six (85.7%) pharmacists, pharmacy knowledge and skills upgrade sessions should be conducted every six months, predominately focussing on software training and inventory management techniques. Communication and documentation skills should be concentrated upon as per 4 (57.1%) and 3 (42.8%) pharmacists respectively.

Doctors can help improve hospital pharmacy management by suggesting alternative drugs in case of non-availability, as per the opinions of 6 (85.7%) pharmacists, and 3 (42.8%) of them felt that effective communication was needed between doctors and pharmacists, especially for suggesting alternative drugs. 

The pharmacy of our institute dispensed 478 formularies of drugs during the study period of 2022-23, and the total annual drug expenditure (ADE) incurred on them was Indian Rupees〈₹〉1,18,81,520. ABC analysis revealed that a category comprising 109 (22.8%) expendable drugs consumed 69.98% of the ADE ₹ 83,14,857. Category B was represented by 115 (24.06%) drugs amounting to 20.0% of the ADE ₹ 23,69,571, and 254 (53.14%) of drugs belonged to category C, consuming only 10.07% ₹ 11,97,092 of the ADE (Table 2).

**Table 2:** Drug items categorized as ABC, VED, and ABC-VED matrix with cost represented as percentage scale for ADE. ABC: always better control, VED: vital essential desirable, ADE: annual drug expenditure

Category	% of items	% of ADE
A (Always)	22.80	69.98
B (Better)	24.06	20.00
C (Control)	53.14	10.07
V (Vital)	6.82	10.87
E (Essential)	60.56	63.62
D (Desirable)	32.62	25.51
I	26.10	71.52
II	55.85	23.84
III	17.99	4.64

The findings of the VED analysis revealed that 33 (6.82%), 289 (60.56%), and 156 (32.62%) drugs belonged to V, E and D category drugs, respectively, amounting to ₹ 12,91,521 (10.87%), ₹ 75,59,023 (63.62%) and ₹ 30,30,976 (25.51%) of total ADE respectively (Table 2).

ABC-VED matrix analysis

**Figure 1:**
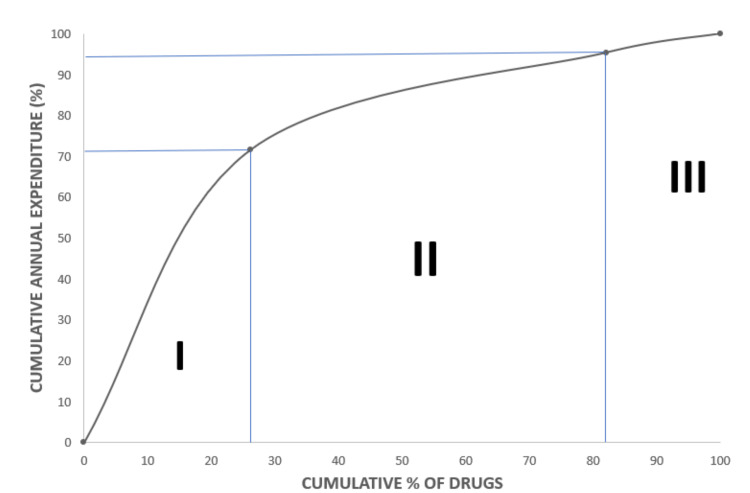
ABC-VED matrix-based categories presented as cumulative percentages against the annual expenditure ABC: always better control, VED: vital essential desirable

There were 125 (26.10%) drugs in category I, 267 (55.8 %) drugs in category II, and 86 (17.99%) drugs in category III, amounting to 71.52% (₹ 84,97,663), 23.84% (₹ 28,32,554) and 4.64% (₹ 5,51,303) of ADE of the pharmacy, respectively (Table 2, Figure 1).

## Discussion

The present study indicates that a newly established pharmacy faces multiple challenges in inventory management. The pharmacy of any hospital consumes a large amount of funds for procurement action and maintenance. Pillans et al. found that better inventory control techniques brought about 20% savings in hospital expenditures [8]. Multiple published studies have shown that making inventory control techniques a routine healthcare practice substantially improves patient care and facilitates optimal use of resources [9,10,11]. Our institute has been established to provide tertiary care healthcare services in a rural part of our country. Because of limited resources and unlimited demands, optimal resource utilization becomes essential.

The challenges we identified for inventory management were issues related to overstocking and expiring a few drugs, leading to expired and obsolete inventory, storage, spacing issues, stockouts, and shortages of others. Knowledge gaps were identified in inventory tracking and technology integration, while communication breakdowns led to supply chain complexities due to the rural location.

The proposed solutions to strengthen the inventory control mechanism were strict adherence to standard operating procedures (SOPs) for drug procurement, storage, dispensing, and inventory control. Effective utilization of the inventory management software will help us to track stock levels, monitor expiration dates, generate reports, and automate the re-ordering process. Regular inventory audits and categorizing medications based on therapeutic class, usage frequency, and storage requirements will help identify discrepancies, detect potential theft or wastage, and ensure adequate inventory data. We plan to integrate the JIT inventory management to reduce the risk of medication expiry, maximize space utilization, optimize cash flow, and reinforce the FIFO approach to ensure that the older inventory is used first or dispensed before the newer stock.

Utilizing barcode technology will help reduce manual errors and improve accuracy in stock management. Periodically categorizing drugs based on ABC and VED analysis assists in forecasting demand, usage patterns, and seasonal variations by analyzing historical data. Maintaining open communication with the vendors and establishing solid partnerships will let us negotiate favorable terms, including delivery schedules, product quality, and pricing. We plan to conduct half-yearly knowledge and skills upgradation sessions for the pharmacy staff, focussing on inventory management techniques, proper documentation, software skills, streamlining operations, stock handling, adherence to SOPs, and communication skills.

Doctors play a crucial role in pharmacy inventory management, and the division of drugs into ABC and VED categories is based on their prescriptions. There are several ways in which doctors can make a significant contribution to the hospital's pharmacy inventory management. Rational and accurate prescriptions, ensuring formulary compliance, and adhering to antimicrobial stewardship will help prevent errors in the dispensing of medications and provide the stocking of necessary drugs. Integration of patients’ electronic health records by the doctors with the pharmacy inventory management systems can assist both doctors and pharmacists to have real-time access to information on drug availability, allowing them to make informed decisions about prescriptions and inventory control. There should be collaborative communication with the pharmacists for updated guidelines, resistance development in the community, and, in the event of drug recalls and shortages, to find suitable alternatives for patients. Doctors must adhere to regulations and prescription guidelines for controlled substances to prevent diversion and ensure proper inventory control.

Healthcare managers need to utilize scientific inventory management methods as the role of a structured hospital organization must be considered. By utilizing selective techniques using various criteria, selected hospital inventory control can be achieved, thereby striking the desired balance between overstocking and stockout situations for superior operational efficiency.

ABC analysis of drugs dispensed during the financial year 2022-23 revealed that 22.80% (109) drugs consumed 69.98 % (category A), and 24.06 % (115) drugs consumed 20 % of the ADE (category B). The remaining 53.14 % (254) drugs consumed only 10% of the ADE (category C). Category A requires strict managerial control, accurate data-driven demand prediction, scrutiny of budgetary control, minimum safety stock, staggered purchase orders, frequent stock-taking, and rational purchasing, stocking, issue, and assessment policies [12,13]. Category B drugs require moderate control by middle-level managers, whereas category C requires minimum control measures by lower-level managers for order and purchase. Selective inventory control should integrate the dual concept of usage and criticality for better management, rather than ABC analysis, which focuses only on annual usage value [14,15].

VED analysis of the drugs during the same period revealed that vital drugs (V) accounted for 6.82 % (33) and essential drugs (E) accounted for 60.56 % (289) of the total inventory, while desirable drugs accounted for 32.62 % (156). Continuous availability and appropriate safety stock should be ensured for vital medicines with zero tolerance for stockout options. Essential drugs can be adjusted to lower resource levels with the availability of substitute drugs. Desirable groups of drugs require little managerial control over their availability and stocking decisions [16].

A combination of ABC and VED analysis revealed that 71.52 % of total ADE was done on 125 (26.1%) category I drugs, which are either expensive or vital, thereby requiring strict managerial control. These drugs should always be maintained in stock as they are crucial. Considering their higher cost, a low buffer stock should be held for them to prevent the locking up of capital. Strict control should be exerted on their prescription and utilization, and a two-bin ordering method needs to be followed. Middle managerial level supervision is adequate for category II (55.85 %) drugs, as they are of intermediate value in monetary terms (23.84 %) and their criticality towards patient care [17]. They can be ordered once or twice a year, saving on ordering costs and reducing management hassles at a moderate carrying cost without blocking substantial capital. Category III (17.99 %), which consumed only 4.64 % of the ADE, can also be ordered once or twice a year, thus reducing ordering costs at a modest carrying cost [18].

Although the present study did not show a significant deviation from previously reported studies discussed above, one of the critical findings of our research is that, out of the total drugs inventory, the vital drugs share for the study period was only 6.82% and accounted for only 10.87% of the ADE. This low contribution of vital medicines to the ADE and issues related to their over-stocking and expiry is because our institute has yet to deliver entire ranges of tertiary care services, and there are currently only two functional OTs. Rationalized stocking of such drugs, especially with short expiry dates, might help in the initial days of hospital services.

The present study is limited because the current expenditure is minimal as the services in critical areas are yet to be started. The spending on vital drugs and category I drugs are bound to increase when the entire twenty OTs become operational and will begin delivering trauma and emergency care, advanced imaging and oncology services, interventional radiology, cardiac catheterization laboratory services, full-fledged planned, different specialty and super specialty critical care services, etc. The other limitation of the study is we have analyzed the ABC, VED, and ABC-VED matrix for the expenditure incurred only for drugs; consumables and equipment cost constitutes a considerable portion that was not included. 

Our participant number was low as it is a single-center survey. Usually, the pharmacist's manpower does not exceed a single-digit count in one institute. Surveying multiple institutes might increase the number of participants. However, the problems faced might not reflect the actual scenario of a rural setup. Finally, volunteer bias cannot be ruled out in such studies. 

## Conclusions

The present study exploring the different pharmacy inventory management challenges remedial measures indicates that the supply chain management faces multiple issues. Adopting bar code technology, integrating patients' electronic health records with the pharmacy inventory management, conducting training sessions focussing on pharmacists’ knowledge and skills, and upgrading every six months will help as a remedial strategy. Using systematic, selective, and scientific inventory control methods like ABC and VED analysis in the early phases of the institute's pharmacy development might help identify the crucial items and areas that need better planning and prioritizing to serve patients better. It might also help in rationalizing the stocking of the drugs with short expiry and used less frequently, even though vital. Doctors might also play an essential role in the inventory management of any hospital.

## Appendices

Appendix 1

Questionnaire on pharmacy management

Pharmacist’s General Profile

Age/Sex - ………………                                                        Position - ………………………..   

Experience in this institute - ……………..

Problems                                                               

What challenges or issues are most frequently encountered in our hospital's pharmacy inventory management?

a.      Overstocking

b.      Understocking

c.      Expiry of medications

d.      Supplier-related problems

e.      Any other …………………

What is the leading cause of pharmacy-related issues in our hospital?

a.      Staffing shortage

b.      Communication-related

c.      Costing related

d.      Technology related

e.      Any other ……………………

3.      Do you need help with stockouts or shortages in our pharmacy?

a.      Regularly

b.      Often

c.      Occasionally

d.      Rarely

e.      Never    

4.      What do you think is the main reason for these delays?

a.      Rural location

b.      Vendor-related issues

c.      Product quality

d.      Cost related

e.      Any other ……………………….

Solutions

5.      Which inventory control method is effective in pharmacy inventory management?

a.      ABC analysis

b.      VED analysis

c.      First-in first out (FIFO)

d.      Just-in-time (JIT)

e.      Any other ……………………….

6.      How relevant is integrating bar code technology with the pharmacy's inventory management system?

a.      Highly relevant

b.      Quite relevant

c.      Somewhat relevant

d.      Not at all relevant

7.      How relevant is integrating Patients' electronic health records with the pharmacy's inventory management system?

a.      Highly relevant

b.      Quite relevant

c.      Somewhat relevant

d.      Not at all relevant

8.      How often should we conduct pharmacy knowledge and skills upgrade sessions for the pharmacy staff?

a.      Every three months

b.      Every six months

c.      Every year

d.      As and when required

9.      What are the necessary training skills that we should focus on?

a.      Inventory management techniques

b.      Documentation

c.      Software training

d.      Communication skills

e.      Any other ……………………….

10.  How can doctors help improve the pharmacy management of a hospital?

a.      Writing accurate and legible prescriptions

b.      Suggest alternative drugs during non-availability

c.      Timely and effective communication

d.      Integration of patients’ electronic health records

e.      Any other ………………………….
